# CD38+ NK cells: novel players in immunoregulation

**DOI:** 10.3389/fimmu.2026.1757958

**Published:** 2026-05-29

**Authors:** Xiaotian Chang, Kehua Fang

**Affiliations:** 1 Medical School of Qingdao Huanghai University, Qingdao, Shandong, China; 2 Department of Emergency and Critical Care Medicine, Qingdao Municipal Hospital (Qingdao Hospital, University of Health and Rehabilitation Sciences), Qingdao, Shandong, China; 3 Clinical Laboratory of The Affiliated Hospital, Qingdao University, Qingdao, Shandong, China

**Keywords:** CD16, CD38, CD38+ NK cells, immune surveillance, immune tolerance, NK cells, regulatory T cells (Tregs), tumor-associated macrophages (TAMs)

## Abstract

CD38 is a transmembrane protein and ectoenzyme that mainly degrades nicotinamide adenine dinucleotide (NAD^+^). Studies have revealed increased numbers of CD38-expressing NK (CD3-CD38+CD56+) cells in many diseases. CD38+ NK cell proportions in the peripheral blood and synovial fluid are increased in patients with rheumatoid arthritis (RA), and these cells produce high levels of interferon-γ (IFN-γ) and low levels of transforming growth factor-β (TGF-β), suppressing the differentiation of CD4+ T cells to regulatory T cells (Tregs) to disrupt immune tolerance. CD38+ NK cell proportions in the peripheral blood and tumor tissues are also increased in patients with colorectal cancer (CRC). However, CD38+ NK cells produce low levels of IFN-γ and NAD^+^ and high levels of TGF-β and adenosine (ADO) and can promote Treg differentiation and macrophage polarization to tumor-associated macrophages (TAMs) to interrupt immune surveillance. CD38+ NK cells were not detected in CD38-KO tumor-bearing mice, and their xenograft tumors grew slowly. Furthermore, the expression of heat shock 70-kDa protein 1B (HSPA1B), a known tumor suppressor, was decreased in CD38+ NK cells from CRC patients but increased in the NK subset from RA patients. HSPA1B can suppress the signaling activity of NF-κB, a regulator of proinflammatory cytokine production. CD38 and CD16 cooperate on the NK cell membrane; most CD38+ NK cells are CD38+CD16+ NK cells that can suppress Treg differentiation. The proportion of CD38+CD16- NK cells among CD38+ NK cells in the peripheral blood was increased in patients with CRC or other tumors. The above results suggest that CD38+CD16+ and CD38+CD16- NK cells have opposing regulatory effects on CD16, HSPA1B and NF-κB signaling and cytokine secretion, leading to opposing effects on immune balance. This review provides a reference for understanding disrupted immune tolerance and surveillance, though the evidence is preliminary.

## 1 Introduction

NK cells are important part of the innate immune system. These lymphocytes can recognize tumor cells and infected cells and exhibit natural cytotoxicity. NK cells also participate in the regulation of the immune response, secrete cytokines, such as interferon-gamma (IFN-γ), and alter the activity of immune cells (1–3). CD38 is a transmembrane protein and ectoenzyme that catalyzes the degradation of nicotinamide adenine dinucleotide (NAD^+^) to adenosine diphosphate ribose (ADPR) and cyclic ADP-ribose (cADPR). ADPR and cADPR are potent Ca^2+^ mobilizers that control calcium flow in the cytoplasm. CD38 also functions as a receptor, conveying transmembrane signals and modifying cell–cell interactions (4). CD38 was first detected in T cells, dendritic cells, NK cells and plasma cells in the 1990s (5). In 1999, Sconocchia et al. reported that CD38 triggered cytotoxic responses in activated human NK cells. Since then, CD38+ NK (CD3-CD38+CD56+) cells have been formally recognized as a subset of NK cells (6).

## 2 Biology of CD38+ NK subsets

Since the 2000s, CD38+ NK cells have been detected in many diseases. A high proportion of CD38_bright_CD69+ NK cells was found in HIV-infected infants (7). The proportion of CD38+CD39+ NK cells was also found to be significantly associated with the progression of HIV infection (8). Moreover, an increased proportion of FcϵRIγ+CD38+CD16_low_+CD56_low_+ NK cells was detected in HIV-exposed uninfected infants at one month postpartum (9). Additionally, an increased proportion of CD38+CD11a+CD95+ NK cells was detected in patients with melanoma compared with that in healthy controls (10). An increased proportion of human leukocyte antigen DR (HLA-DR)+CD38+ NK cells was detected in patients with biliary atresia (11), increased proportions of CD38+CD73+ NK cells were detected in patients with genotypes 1 and 3 of chronic viral hepatitis C at the fibrosis stage (12), and NK cells with increased CD38 expression were detected in pregnant women. CD38 and NK cell p46-related protein (NKp46) are considered the best markers for distinguishing and separating the NK cells of pregnant women from those of postpartum women (13). CD38 expression was higher in the NK cells of coronavirus disease 2019 (COVID-19) patients than in those of healthy donors (14), and the proportion of CD38+CD56_dim_ NK cells with killer cell lectin like receptor G (KLRG-1), CD64, CD15 and CD197 expression was decreased in severe chronic kidney disease patients (15). Our group reported significantly increased proportions of CD38+ NK (CD3-CD38+CD56+) cells in the peripheral blood, synovial fluid and synovial tissues of patients with rheumatoid arthritis (RA). The proportion was strongly associated with the simple disease activity index (SDAI), a clinical indicator for the diagnosis of RA (16, 17). Additionally, CD38 expression was increased in the NK cells of patients with systemic lupus erythematosus (SLE) (18). A summary of the disease associations of CD38+ NK cells is provided in **Table 1**. Although different studies applied different gating strategies to measure the proportions of CD38+ NK cells and CD38+ NK cells exhibit differential expression of cell surface markers, these NK cells have high CD38 expression and are present at high numbers in association with disease. Additionally, CD11a, CD15, CD16, CD39, CD64, CD69, CD73, CD95, CD197, NKp46 and KLRG-1 possibly cooperate with CD38 to function in NK cells.

**Table 1:** Disease associations of CD38+ NK cells.

CD38+ NK cells	Disease/condition	Level	Gating principles	References
CD38_bright_ CD69+ NK	HIV-infected infants	increased	CD3- CD16+ CD38+ CD56+ CD69+	(7)
CD38+CD39+ NK	HIV infection	increased	CD3- CD14- CD16+ CD19- CD38+ CD56+ CD39+	(8)
CD38+CD56_low_ CD16_low_+FcϵRIγ+ NK	HIV-exposed uninfected infants	increased		(9)
CD38+CD11a+CD95+ NK	melanoma	increased	CD11a+ CD16+ CD38+ CD56+ CD95+	(10)
HLA-DR+CD38+ NK	biliary atresia	increased	CD3- CD38+ CD56+ HLA-DR+	(11)
CD38_hi_+ NK	pregnant women		CD3- CD14- CD16+/- CD19- CD20- CD56+	(13)
KLRG-1+CD38+CD64+CD15+CD197+ NK	chronic kidney disease	declined	KLRG-1+ CD15+ CD38+ CD64+ CD197+	(15)
CD38_hi_+ NK	COVID-19 infection		CD38+ CD56+ CD16+	(14)
CD38+CD73+ NK	genotypes 1 or 3 chronic viral hepatitis C	increased	CD38+ CD56+ CD73+	(12)
CD3-CD38+CD56+ NK	rheumatoid arthritis	increased	CD3- CD16+ CD38+ CD56+	(16)
CD38_hi_+ NK	systemic lupus erythematosus		CD3- CD7+ CD14- CD16+/- CD19- CD56+	(18)
CD38+ NK	multiple myeloma		CD3- CD38+ CD56+	(19)
CD38+ NK	colorectal cancer	increased	CD3- CD38+ CD56+	(20)
CD38+ NK	breast cancer	increased	CD3- CD38+ CD56+	(20)
CD38+ NK	gastric cancer	increased	CD3- CD38+ CD56+	(20)
CD38+ NK	esophageal cancer	increased	CD3- CD38+ CD56+	(20)
CD38+ NK	ovarian cancer	increased	CD3- CD38+ CD56+	(20)
CD38+ NK	lung cancer	increased	CD3- CD38+ CD56+	(20)
CD38+CD16+/-CD56 NK	gastric cancer	increased	CD3- CD16+/- CD38+ CD56+	(20)
CD38+CD16+/-CD56 NK	esophageal cancer	increased	CD3- CD16+/- CD38+ CD56+	(20)
CD38+CD16+/-CD56 NK	ovarian cancer	increased	CD3- CD16+/- CD38+ CD56+	(20)
CD38+CD16+/-CD56 NK	lung cancer	increased	CD3- CD16+/- CD38+ CD56+	(20)

CD38 mainly metabolizes NAD^+^, and decreased NAD^+^ levels are associated with metabolic and inflammatory diseases, aging and neurodegenerative disorders (5). CD38+ NK cell proportions may be altered during many infectious diseases and chronic inflammatory diseases, as reported in the above studies. However, few studies investigated the function of CD38+ NK cells at that time. One study reported that treatment with anti-CD38 antibodies could activate the release of granzymes and change cytokine production by NK cells, suggesting that CD38 regulated cytotoxic responses and cytokine secretion in NK cells (6).

## 3 CD38+ NK cells in autoimmune diseases (rheumatoid arthritis and systemic lupus erythematosus)

Immune tolerance refers to the unresponsive state of the immune system to specific antigens. Failure or breakdown of immune tolerance results in autoimmunity. In autoimmune diseases, the immune system mistakenly recognizes normal cells and tissues as foreign substances and attacks them (21). CD4+ T cells can develop into regulatory T cells (Tregs), T helper (Th) 1 cells, Th17 cells, and Th2 cells. Tregs facilitate immunological tolerance and suppress excessive immune responses (22, 23). RA is an autoimmune disease that is characterized by chronic joint inflammation. Functional dysregulation and decreased proportions of Tregs contribute to immune imbalance and autoimmunity in patients with RA and other autoimmune diseases. An increase in RA activity is strongly correlated with a considerable decrease in the Treg fraction in patients (24, 25). Clinical trials have shown that injection of Tregs is an effective treatment for autoimmune diseases such as inflammatory bowel disease and multiple sclerosis (26–28). We injected human-derived Tregs into rats with collagen-induced arthritis (CIA), which is a commonly used animal model for RA research, and demonstrated that cross-species Treg infusion had immunosuppressive and anti-inflammatory effects on CIA (29). However, the mechanism underlying decreases in Treg proportions in patients with RA and other autoimmune diseases is poorly understood.

Many studies have reported increased CD38 expression in patients with RA. CIA was significantly alleviated in CD38-knockout mice (30, 31). Injecting cynomolgus monkeys with anti-CD38 antibodies significantly prevented CIA development (32). High percentages of CD38+ NK (CD3-CD38+CD56) cells were detected in the peripheral blood and synovial fluid of CIA mice. Increased proportions were also detected in the peripheral blood and synovial fluid of RA patients. Moreover, an increase in the CD38+ NK cell proportion was negatively associated with a decrease in the Treg proportion (33). SLE is an autoimmune disease that affects many systems. CD38 expression is also increased in the NK cells of patients with SLE (18).

To investigate how CD38+ NK cells affect immune tolerance, CD38+ NK cells and mononuclear cells (MNCs) depleted of CD38+ NK cells were sorted from the peripheral blood of RA patients by magnetic bead separation. These CD38+ NK cells were cocultured with MNCs in separate Transwell chambers. The percentage of Tregs was significantly decreased among the cocultured MNCs. The naturally occurring CD38 inhibitor cyanidin-3-O-glucoside (C3G) competitively binds to the active site of CD38 to block its activity (34); thus, it was hypothesized that C3G could ameliorate RA through the suppression of CD38 activity. As anticipated, in CIA rats that received C3G therapy, joint inflammation was significantly alleviated, and Treg levels were elevated in the peripheral blood and synovial fluid. Furthermore, the percentage of Tregs was greater among MNCs that were cocultured with C3G-pretreated CD38+ NK cells than among those cocultured with PBS-pretreated CD38+ NK cells. Hence, the decrease in the percentage of Tregs among MNCs may be due mainly to the CD38+ NK cell-mediated suppression on Treg differentiation (33). Compound 78c is a potent CD38 chemical inhibitor with favorable tissue uptake (35, 36). Following 78c treatment, CIA was significantly alleviated in the mouse model, and the Th17 cell/Treg and Th1/Th2 cell ratios in peripheral blood decreased. The percentage of CD38+ NK cells was also decreased in the animal model. Compared with noncocultured CD4+ T cells, those that were cocultured with 78c-pretreated CD38+ NK cells from healthy people could differentiate into more Tregs and had lower Th17 cell/Treg and Th1/Th2 cell ratios (37). The results of the above two experiments using C3G and 78c suggested the inhibitory role of CD38+ NK cells on Treg differentiation. One group revealed that the proportions of CD38+CD39+ NK cells in HIV-infected individuals were negatively associated with the CD4+ T-cell counts and suggested that targeting CD38 and CD39 on NK cells might be a potential therapeutic strategy against HIV infection (8). Their results suggest that CD38+ NK cells may affect Treg differentiation by regulating not only the differentiation but also the proliferation of CD4+ T cells.

Sirtuins (Sirts) are a family of NAD^+^-dependent deacetylases that regulate cellular metabolism, stress responses, aging and disease processes. CD38 suppresses sirtuin activity; therefore, the inhibition of CD38 activity is an effective way to promote sirtuin activation (38, 39). Sirt6, a member of the sirtuin family, is a deacetylase that requires NAD^+^. A Sirt6-harboring adenovirus has been demonstrated to lessen tissue damage and inflammatory responses in CIA animals (40). In contrast to that among MNCs that were cocultured with CD38+ NK cells pretreated with C3G alone, the Treg percentage remained low among MNCs that were cocultured with CD38+ NK cells pretreated with both C3G and a Sirt6 siRNA. Additionally, Sirt6 expression was elevated in CD38+ NK cells following C3G or 78c treatment, regardless of whether these NK cells were extracted from the peripheral blood of individuals with RA or healthy people (33, 37). The results of the above experiments suggest that CD38 suppresses Treg differentiation by downregulating Sirt6 expression or activity in CD38+ NK cells and that C3G- or 78c-mediated blockade of CD38 expression increases Sirt6 expression and facilitates Treg differentiation (33, 37). Another study reported that treatment with an anti-CD38 biparatopic antibody inhibited CD38 enzymatic activity, which in turn increased intracellular NAD^+^ levels and Sirt1 and Sirt3 activity (41). The finding suggests that Sirt1 and Sirt3 are also involved in the CD38-regulatory pathway.

To further understand the immunoregulatory mechanism of CD38+ NK cells in Treg differentiation, the production of proinflammatory cytokines by CD38+ NK cells was investigated. Decreased IFN-γ levels and high tumor necrosis factor-alpha (TNF-α) levels were detected in the culture medium of CD38+ NK cells following C3G treatment. MNCs cocultured with CD38+ NK cells showed an increase in the number of Tregs when the NK cells were pretreated with an anti–IFN-γ antibody or TNF-α, but the number of Tregs decreased when IFN-γ or an anti–TNF-α antibody was used. However, combined treatment with C3G and a Sirt6 siRNA did not affect IFN-γ or TNF-α secretion by CD38+ NK cells (33). Low IFN-γ and IL-17A levels were also detected in the coculture medium of CD4+ T cells and 78c-pretreated CD38+ NK cells (37). Additionally, compared with that of MNCs and PBS-pretreated CD38+ NK cells, the coculture medium of MNCs and CD38+ NK cells that were pretreated with an anti-CD38 antibody contained low interleukin-6 (IL-6) and IFN-γ levels and high interleukin-2 (IL-2) and interleukin-10 (IL-10) levels (42). CD38 has been reported to stimulate IFN-γ release by NK cells (43), and Sirt6 enables the release of TNF-α (44). Many studies have verified that IFN-γ can block Treg differentiation and that TNF-α can facilitate Treg activation and proliferation (45–47). Activated NK cells release more IFN-γ to suppress forkhead box P3 (Foxp3) transcription in Tregs (48). The results of the above studies suggest that CD38+ NK cells inhibit CD4+ T-cell differentiation into Tregs by regulating proinflammatory cytokine secretion.

Although IL-2 alone cannot induce Treg differentiation, IL-2 in combination with transforming growth factor-beta (TGF-β) can induce Treg differentiation (49). Furthermore, the combination of interleukin- 21 (IL-21) or IL-6 with TGF-β can drive the differentiation of naïve CD4+ T cells into Th17 cells, and TGF-β can stimulate the differentiation of CD4+ T cells into Tregs (50, 51). *In vitro*, CD4+ T-cell differentiation into Tregs can be induced by anti-CD28, anti-CD3, TGF-β and IL-2 (47). When CD38+ NK cells from the peripheral blood of RA patients were cocultured with CD4+ T cells from healthy people, pretreatment of CD38+ NK cells with an anti-CD38 antibody increased the production of TGF-β but not the expression of CD28 or CD3 (42). RA has been linked to low serum TGF-β levels (52, 53). On the basis of the results of our study and other studies, it is possible that CD38+ NK cells, which are present in high proportions in RA patients, secrete low levels of TNF-α, TGF-β and IL-2 and high levels of IFN-γ through the CD38–Sirt6 pathway to suppress Treg differentiation.

The mammalian target of rapamycin (mTOR) cascade blocks Treg expansion (54, 55), and researchers have developed mTOR cascade-suppressing agents to prevent and treat RA by increasing the Treg count (56, 57). Following coculture with CD38+ NK cells, CD4+ T cells showed markedly higher phospho-mTOR, phospho-P70S6, and total mTOR protein levels than did CD4+ T cells that were not cocultured. Following coculture with CD38+ NK cells that were pretreated with an anti-CD38 antibody, CD4+ T cells showed lower phospho-mTOR, phospho-P70S6 and total mTOR protein levels than did CD4+ T cells cocultured with PBS-pretreated CD38+ NK cells (42). In SLE, mTOR activation also induces the activation of CD4+ T cells, skewing their differentiation towards Th17 cells and resulting in increased Th17/Treg cell proportions (58). These observations suggest that coculture with CD38+ NK cells activates the mTOR cascade in CD4+ T cells to block Treg differentiation. Many studies have shown that IFN-γ suppresses the differentiation of CD4+ T cells into Tregs through the activation of mTOR, and that TGF-β, IL-2 and TNF-α have opposite effects. For example, TGF-α1 preserved Foxp3 expression in Tregs by suppressing mTOR signaling (59); TGF-β1 stimulated the mTOR cascade during M2-type macrophage polarization (60); TNF-α signaling promoted metabolic reprogramming in CD4+ T cells through the mTOR axis (61); IL-2 was associated with an upstream pathway of the mTOR cascade (62); and IFN-γ induced high mTOR activity in macrophages, lung epithelial adenocarcinoma cells and Paneth cell death (63–65). These studies suggest a possibility that CD38+ NK cells regulate cytokine production to induce Treg differentiation by mediating mTOR signaling in CD4+ T cells.

A transcriptomic analysis was performed to investigate the mRNA expression profiles of CD38+ NK cells that were cocultured with CD4+ T cells. To elucidate the downstream regulatory mechanism of CD38, CD38+ NK cells were pretreated with 78c to inhibit CD38 expression and activity. KEGG enrichment analysis revealed that in CD38+ NK cells from patients with RA, CD38 was associated with pathways related to rheumatoid arthritis, TNF signaling, IL-17 signaling, the hematopoietic cell lineage, complement and coagulation cascades, the NF-κB cascade, osteoclast differentiation, and cytokine–cytokine receptor interactions. These pathways are well known to be involved in RA pathogenesis and autoimmune diseases.

**Figure 1:**
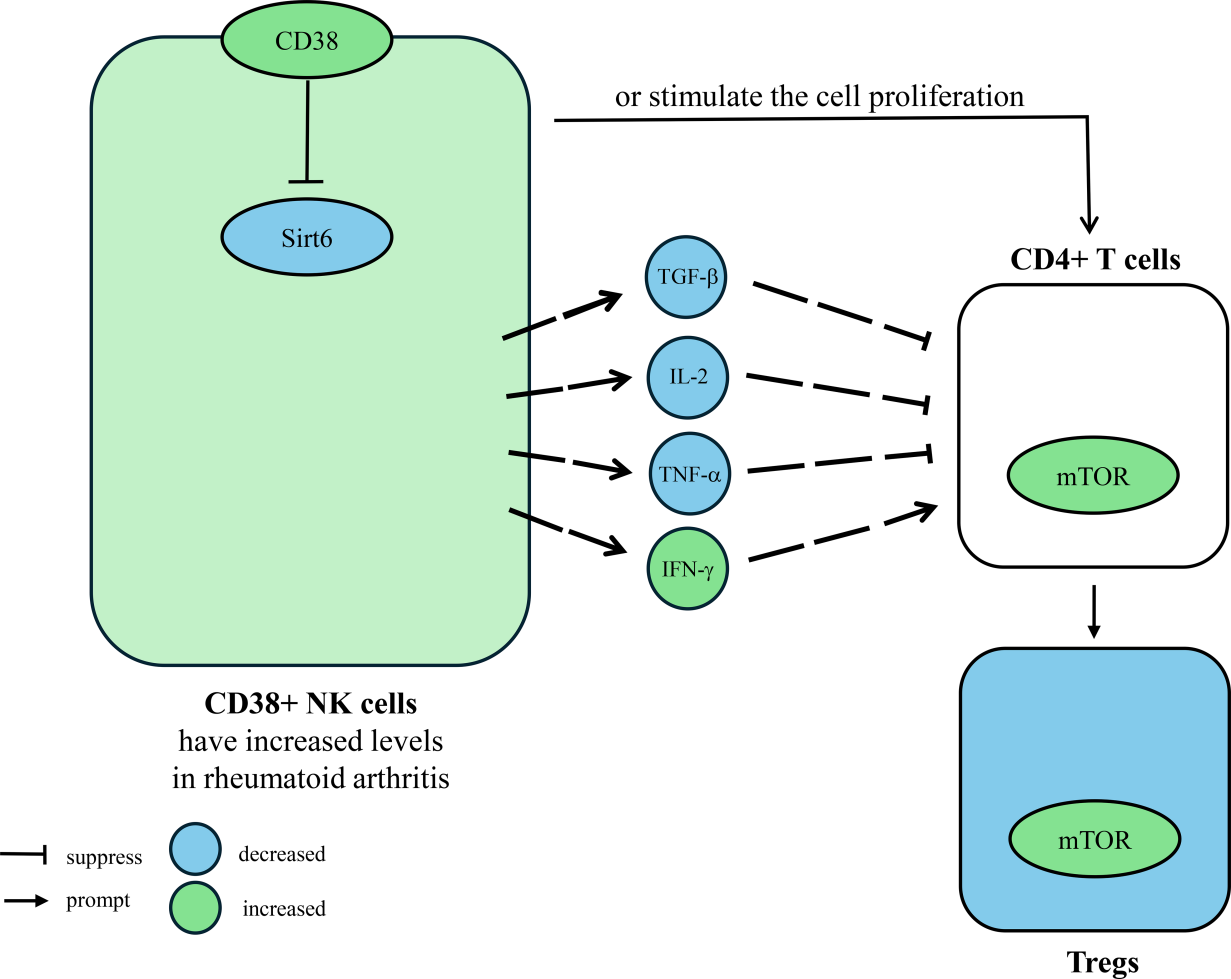
Potential mechanism through which CD38+ NK cells regulate Treg differentiation in patients with rheumatoid arthritis. In CD38+ NK cells, a high CD38 level suppresses Sirt6 expression, which increases IFN-γ production and decreases TGF-β, IL-2 and TNF-α levels to prevent CD4+ T-cell differentiation into Tregs.

The data in **Figure 1** suggest a possible role for CD38+ NK cells in Treg differentiation in RA. Such effects may also exist in other autoimmune diseases, such as SLE, because disruptions in Treg function or abundance disturb immune tolerance in autoimmune diseases (66).

## 4 CD38+ NK cells in cancers (colorectal cancer, multiple myeloma, melanoma and other tumors)

Immune surveillance has a systemic inhibitory effect on tumor development. High levels of adenosine (ADO), programmed death 1 (PD-1)/programmed cell death-ligand 1 (PD-L1), Tregs and tumor-associated macrophages (TAMs) can disrupt immune surveillance (67, 68). Although CD38 is overexpressed in many cancer cells (69), CD38+ NK cells have been reported mainly in multiple myeloma (MM), the common hematologic malignancy. The CD38+ NK subset has rarely been reported in solid tumors. The proportions of CD38+ NK cells in the peripheral blood of tumor patients were recently measured. The percentages of CD38+ NK cells among total lymphocytes were markedly increased, by approximately 20% to 30%, in patients with colorectal cancer (CRC), breast cancer (BC), gastric cancer (GC), esophageal cancer (EC), ovarian cancer (OC) and lung cancer (LC) compared with those in healthy people (20). Immunohistochemistry and immunofluorescence also revealed a high abundance of CD38+ NK cells in CRC tumor tissues (70). Moreover, CRC patients with positive lymph node metastasis have a considerably high proportion of CD38+ NK cells in the peripheral blood, and a high percentage of circulating CD38+ NK cells is positively related to poor CRC prognosis (71). Furthermore, an increased proportion of CD38+ NK cells was detected in the blood of tumor-bearing C57BL/6J mice that were injected with MC38 cells, a mouse colon tumor cell line (20). Therefore, high percentages of CD38+ NK cells may be present in blood and tissues from humans and model animals with cancer. Additionally, CD38+ NK cells derived from the peripheral blood of CRC patients displayed unique expression and metabolic traits, as demonstrated by transcriptional and metabonomic analyses. Compared with the expression profiles of the NK subset from healthy people and NK cells treated with anti-CD38 antibodies, those of CD38+ NK cells from patients with CRC include alternative expressions of genes involved in active chemokine signaling pathways, cytokine–cytokine receptor interaction pathways, the TNF pathway, the IL-17 pathway, the Toll-like receptor signaling pathway, tryptophan metabolism, hematopoietic cell lineage-related pathways, the NOD-like receptor signaling pathway, and the NF-κB pathway (70).

Low NK cell killing capacity is an important immune trait of patients with cancer. The capacity of CRC-derived CD38+ NK cells to kill SW480 tumor cells *in vitro* was reduced by almost 50%. On the other hand, coculture with CD38+ NK cells derived from the peripheral blood of CRC patients increased SW480 cell proliferation by 15% (20). No CD38+ NK cells were detected in tumor-bearing CD38-KO C57BL/6J mice with MC38 cell-derived xenografts, and tumor growth was significantly reduced in the CD38-KO mice compared with wild-type C57BL/6J tumor-bearing animals (20). These observations support the possibility that CD38+ NK cells in CRC favor tumor growth and have low killing activity. Other studies have demonstrated that CD38 expression contributes to a decrease in the killing ability of NK cells in MM (72, 73).

NAD^+^ improves the anticancer immune response by enhancing T-cell responses. Cells undergo epithelial–mesenchymal transition (EMT) when their NAD^+^ concentration decreases (74, 75). NAD^+^ production by CD38+ NK cells derived from CRC patients was only half of that by CD38+ NK cells from healthy individuals (20). CD38 is known to degrade NAD^+^ to produce ADO (76–78). The level of ADO increased by 20% in the culture medium of CRC patient-derived CD38+ NK cells. Metabonomic analysis also confirmed that CRC patient-derived CD38+ NK cells produced more ADO. Additionally, CRC patient-derived CD38+ NK cells exhibited a 15% increase in PD-1 expression (20). It is well known that the PD-1/PD-L1 axis inhibits adaptive immune responses primarily by suppressing effector T-cell function and promoting immunosuppressive Treg activity (79). These results suggest that the high numbers of CRC patient-derived CD38+ NK cells increase ADO secretion and PD-1 expression and decrease NAD^+^ production to promote tumor growth in the TME in CRC.

The primary roles of Tregs and M2-type macrophages, also referred to as TAMs, include their protumorigenic function and stimulation of tumor cell immune escape (80). For most solid tumors, a high M2-type macrophages/M1-type macrophages ratio of macrophages is an indicator of a poor prognosis (81). To observe the effect of CD38+ NK cells on macrophage polarization, M0-type macrophages were induced from THP-1 cells, a human leukemia monocytic cell line, and then cultured with culture medium from CD38+ NK cells. Compared with those cultured in the medium of CD38+ NK cells from healthy individuals, more M0 macrophages that were cultured in the medium of CD38+ NK cells from CRC patients were polarized to M2-type macrophages, so-called TAMs. Additionally, when CD4+ T cells were cultured with CRC patient-derived CD38+ NK cells, the number of Tregs among CD4+ T cells doubled (70). This situation is just the opposite effect that was observed in CD4+T cells cocultured with CD38+ NK cells from RA patients (42). Furthermore, compared with wild-type control mice, tumor-bearing C57BL/6J mice with MC38 cell transplantation had more Tregs and fewer Th1 cells. However, the proportions of Th1 cells and Tregs did not change in CD38-KO animals with tumor engraftment, and no CD38+ NK cells were detected in the KO animals (20). These *in vitro* and *in vivo* results suggest that high CD38+ NK cell levels in CRC patients interfere with immune surveillance by promoting CD4+ T-cell differentiation into Tregs and macrophage polarization into TAMs, which may favor tumor cell immune escape. Considering that CD38+ NK cells are highly prevalent in many cancers including BC, CRC, EC, GC, LC, melanoma and OC (10, 20), comparable conditions could occur in other malignancies.

The production of proinflammatory cytokines was investigated in CD38+ NK cells from tumors. When CD4+ T cells from healthy individuals were cocultured with CD38+ NK cells from CRC patients, IFN-γ levels decreased and IL-10 and TGF-β levels increased in the coculture medium (70). Increased TGF-β levels and decreased TNF-α levels were also detected in the culture medium of CD38+ NK cells from CRC patients compared with those in the medium from CD38+ NK cells from healthy controls. On the other hand, the IFN-γ level increased when CD38+ NK cells were pretreated with anti-CD38 antibodies (20). IFN-γ also activates M1 macrophage polarization and thus increases the M1/M2 macrophage ratio (82). These results are the opposite of those in the RA context and suggest that in CRC, abundant CD38+ NK cells may secrete high amounts of TGF-β and low amounts of IFN-γ to promote CD4+ T-cell differentiation into Tregs and macrophage polarization into TAMs, thus interfering with immune surveillance.

One study reported that the abundance of CD56_bright_ NK cells was negatively correlated with the overall survival of patients with late-stage (III/IV) melanoma. Compared with those from healthy controls, the CD56_bright_ NK cells from the patients exhibited upregulated CD11a, CD38 and CD95 expression. These CD56_bright_ NK cells produced less TNF-α and granulocyte–macrophage colony-stimulating factor (GM-CSF) in patients than in controls. Furthermore, IFN-γ production by these CD56_bright_ NK cells was inversely correlated with the overall survival of patients. This result suggests the presence of a subtype of NK cells that plays an immunomodulatory role and even stimulates tumor growth in melanoma (10). Another study showed the stimulatory effect of uterine NK cells on Treg differentiation, resulting in maternal–fetal tolerance in murine and human pregnancy (83). Furthermore, one recent study detected an increase in the high proportion of CD38+ NK cells in pregnant women (13). NK cells use different strategies to limit T-cell function through not only cytokines but also cellular interactions with NK receptors NKG2D and NKp46 or through perforin-mediated T-cell death (84). NK cells also inhibit the generation of autoreactive T cells, lysing antigen-presenting cells and other regulatory cells by producing regulatory cytokines (85).

Nuclear factor kappa-B (NF-κB) is involved in a proinflammatory cascade that controls cytokine release (86). An opposing Sirt1–NF-κB interaction affects energy metabolism and innate immunity (87). Sirt1 physically interacts with NF-κB and prevents its transcription by deacetylating the NF-κB RelA/p65 subunit in macrophages (88). Substantial 50% decreases in Sirt1 and Sirt6 expression, a 20% increase in NF-κB acetylation, and a twofold increase in NF-κB expression were detected in CRC patient-derived CD38+ NK cells. Anti-CD38 antibody treatment conversely increased Sirt1 and Sirt6 expression but decreased NF-κB acetylation and expression in CD38+ NK cells. These results suggest that increased CD38 activity and expression in CRC patient-derived NK cells decrease Sirt1 and Sirt6 levels, which in turn increases NF-κB expression and acetylation (20, 70). NF-κB has been reported to increase TNF-α expression in myeloid-derived suppressor cells (MDSCs) (89), promote the release of IL-10 in microglia and increase the TGF-β level in endothelial cells (90, 91). MicroRNA-146a regulates IFN-γ production by targeting NF-κB signaling in NK cells (92). A study reported that Sirt6 was a negative regulator of the antitumor function of NK cells in murine colorectal cancer (93). Activation of the NF-κB pathway resulted in the increased secretion of proinflammatory cytokines, such as TNF-α, IL-6 and IL-1β, and the neutrophil chemoattractant MIP-2 in macrophages (94).

Transcriptomic analysis revealed that compared with those from healthy controls, CD38+ NK cells from CRC patients had significantly lower mRNA levels of heat shock protein family A (Hsp70) member 1B (HSPA1B). When CRC CD38+ NK cells were cultured with an anti-CD38 antibody, HSPA1B expression increased. Both real-time PCR and western blot analysis revealed that compared with NK cells from healthy people, CD38+ NK cells from CRC patients had a 50% lower HSPA1B expression level (20). HSPA1B is an apoptosis-related factor and has been reported to promote NK cell proliferation and cytolytic function while increasing IFN-γ secretion (95–97). HSPA1B also activates immune cells to trigger anticancer immune responses (98). In a mouse skin allograft model, disrupted HSPA1B gene expression extended the growth of the graft (99). HSPA1B overexpression can block the degradation of IkappaB α (IκBα), an NF-κB activity regulator, resulting in the suppression of the nuclear translocation of p65 to transcriptionally activate NF-κB signaling (100). In lung tissues, HSPA1B deletion prolonged NF-κB activation (101). HSPA1B regulates the NF-κB pathway via toll like receptor 2 (TLR2) and toll like receptor 4 (TLR4) (94). Therefore, it is possible that CD38 blocks HSPA1B expression and activates NF-κB function in CRC-derived CD38+ NK cells, which in turn influence Treg differentiation and macrophage polarization by regulating IFN-γ and TGF-β production to disrupt immune surveillance. It is unclear whether CD38 blocks HSPA1B expression by downregulating Sirt1, Sirt6 or other sirtuin members or other pathways.

**Figure 2:**
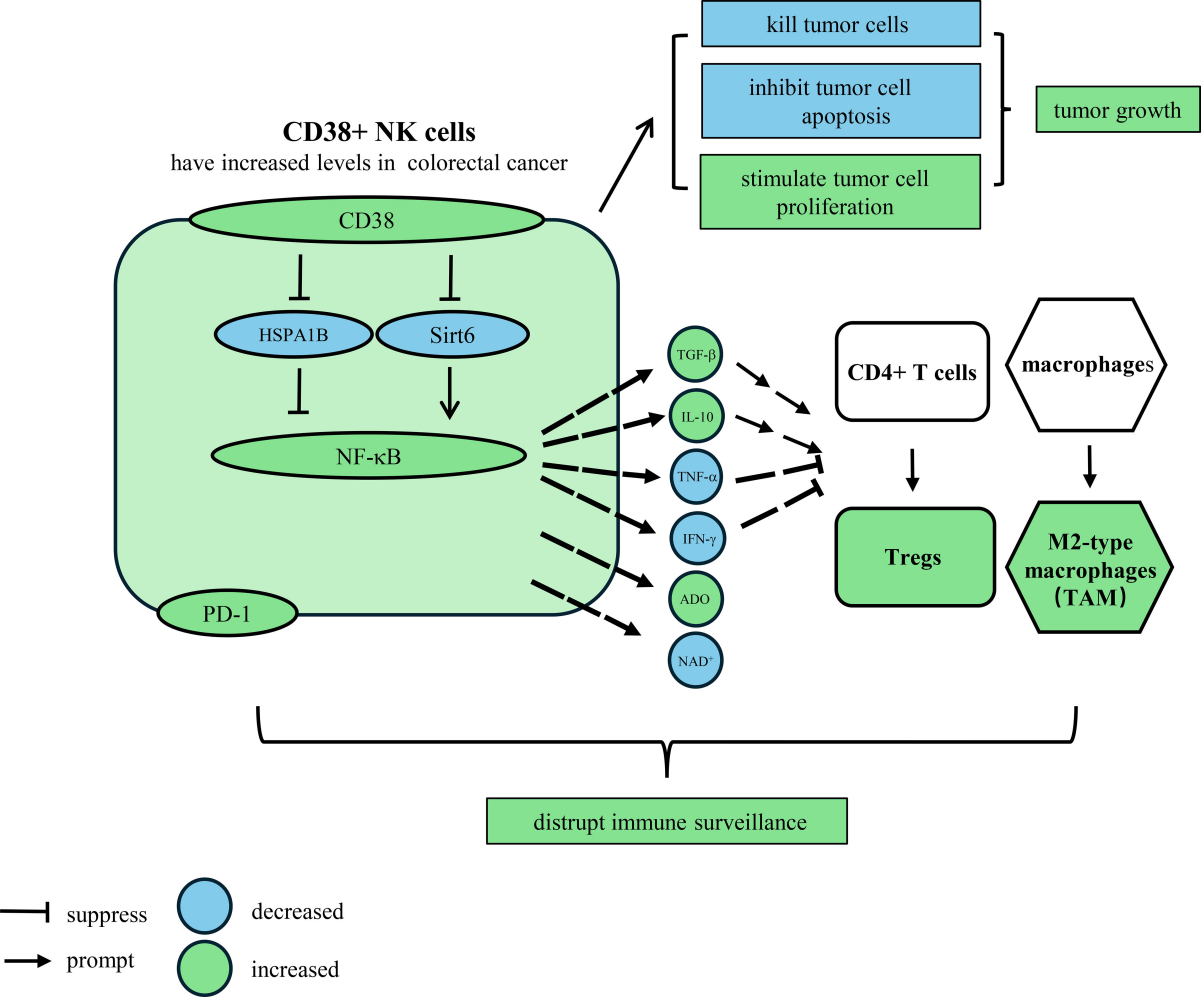
Potential mechanism through which CD38+ NK cells mediate the immune microenvironment in colorectal cancer. High levels of CD38 suppress Sirt6 and HSPA1B expression in CD38+ NK cells, which subsequently increase NF-κB activity to mediate cytokine production. CD38+ NK cells produce high levels of ADO and TGF-β and low levels of NAD+, IFN-γ and TNF-α. As a result, CD38+ NK cells promote CD4+ T-cell differentiation into Tregs and macrophage polarization to the M2 type to alter immune surveillance.

The data in **Figure 2** suggest a possible mechanism involving CD38+ NK cells in CRC. A similar situation is hypothesized to occur in patients with other cancer types, because high numbers of CD38+ NK cells were also detected in the peripheral blood of patients with other cancers.

## 5 CD38+CD16+ and CD38+CD16- NK cells: opposing roles and mechanisms

The percentages of CD38+ NK cells are increased in both RA patients and CRC patients, which raises a question why their immunomodulatory effects lead to opposite outcomes in these two diseases. NK cells consist of two subtypes, namely, CD16+ and CD16- NK cells. Many studies have reported that CD16_bright_CD56_dim_ NK cells are extremely cytotoxic (102) and that CD16-CD56_bright_ NK cells impair the immunological surveillance system (103). In healthy people, CD16_dim_CD56_bright_- NK cells express CD38 on the surface (104), and CD38+ NK cells from healthy people and patients with RA exhibit increased CD16 expression (42). Hence, CD38+ NK cells consist of two subtypes, namely, CD38+CD16+ and CD38+CD16- NK cells, corresponding to CD16+ NK cells and CD16- NK cells, respectively, and even CD16_bright_ CD56_dim_ NK cells and CD16-CD56_bright_ NK cells. CD38+CD16- NK cells are found at higher percentages in the blood of patients with GC, CRC, OC or LC, and the ratios of CD38+CD16- NK cells/CD38+ NK cells and CD38+CD16- NK cells/total lymphocytes are increased in the peripheral blood of these tumor patients, although most CD38+ NK cells are of the CD38+CD16+ NK subset (20). Recent research has also shown that approximately 90% of CD38+ NK cells express CD16 in healthy people as well as in patients with MM. Compared with healthy controls, newly diagnosed patients with MM have relatively lower percentages of CD38+CD16+ NK cells and higher percentages of CD38+CD16- NK cells (105). Moreover, the infiltration of CD56_bright_CD16_dim_/- NK cells is increased in tumor tissues of papillary thyroid tumors, non-small cell lung cancer, and prostate cancer, and the degree of CD56_bright_CD16- NK cell infiltration is linked to the advancement of tumor stages and decreased killing ability (106–109), but whether these CD16- NK cells in tumor tissues express or not CD38 is unknown. On the other hand, compared with osteoarthritis (OA) patients and healthy controls, RA patients have significantly greater percentages of CD38−CD16+CD56+ NK cells in their synovial fluid and peripheral blood (42).

Evidence suggests the inhibitory effect of CD38+CD16+ NK cells on CD4+ T-cell differentiation. CD38+CD16+CD56+ NK cells and CD4+ T cells were isolated from the synovial fluid of RA patients and peripheral blood of healthy individuals, respectively, by using flow cytometry. In contrast to noncocultured CD4+ T cells, CD4+ T cells that were cocultured with CD38+CD16+CD56+ NK cells had increased Th1/Th2 cell and Th17 cell/Treg ratios. CD4+ T cells cocultured with CD38+CD16+CD56+ NK cells that were pretreated with an anti-CD38 antibody had decreased ratios, in contrast to those cocultured with PBS-pretreated CD38+CD16+CD56+ NK cells. Furthermore, compared with that of noncocultured NK cells, the coculture medium of CD38+CD16+ NK cells had reduced IL-10 levels and increased IFN-γ levels. Moreover, compared with PBS-pretreated CD38+CD16+ NK cells, CD38+CD16+ NK cells that were pretreated with an anti-CD38 antibody presented low IL-6 and IFN-γ levels and high IL-2 and IL-10 levels in the culture medium (42). These results suggest that the percentage of CD38+CD16+ NK cells among CD38+ NK cells is increased in RA patients, which contributes to a decrease in the percentage of Tregs through the production of more IFN-γ (42). On the other hand, compared with CD38+ NK cells from healthy people or RA patients, CD38+ NK cells from CRC patients, which contain relatively high proportions of CD38+CD16- NK cells, might produce more ADO, PD-1 and TGF-β and less NAD^+^ and IFN-γ, and these NK cells promote CD4+ T-cell differentiation to Tregs and macrophage polarization to the TAM subtype (20, 70).

A comparison of the results of two KEGG analyses between CD38+ NK cells from RA patients and NK cells from CRC patients (37, 70) revealed that TNF signaling, IL-17 signaling, hematopoietic cell lineage signaling, NF-κB signaling and cytokine–cytokine receptor interaction pathways were enriched in both NK subsets. These results suggest that CD38 may play opposite immunomodulatory roles in these two types of NK cells through different regulatory effects on these signaling pathways.

Many studies, including ours, have revealed that CD38+ NK cells from healthy individuals and cancer or RA patients highly express CD38 and that high CD38 expression inhibits Sirt1 and Sirt6 expression (20, 33). Therefore, the CD38–Sirt1 or CD38–Sirt6 interactions do not seem to explain the functionally opposite effects between CD38+ NK cells in RA patients and the NK subset in CRC patients. On the other hand, NF-κB expression is increased in CD38+ NK cells from CRC patients, whereas HSPA1B expression is decreased (20, 70). In contrast, NF-κB expression is suppressed in CD38+ NK cells from healthy persons, and HSPA1B expression is upregulated in CD38+ NK cells from RA patients (37). Moreover, HSPA1B overexpression prevents the activity of the NF-κB complex (100). Therefore, an increased proportion of CD38+CD16- NK cells among CD38+ NK cells in CRC and decreased HSPA1B expression in the NK subset may result in increased NF-κB expression and activity. Moreover, the inhibitory effect of CD38 on Sirt1 and Sirt6 expression releases the NF-κB activity. In CD38+ NK cells from healthy people, which consist mainly of CD38+CD16+ NK cells, an increase in HSPA1B expression suppressed NF-κB activity, although the inhibitory effect of CD38 on Sirt1 and Sirt6 expression simultaneously abrogated the inhibitory effect of sirtuin members on NF-κB activity (70). To date, there have been no reports on the relationship between Sirt6 and HSPA1B in NK cells. Only one study reported that Sirt1 inhibited the upregulation of HSPA1A and HSPA1B expression in resting B cells (110). However, why HSPA1B expression is high in CD38+CD16+ NK cells and low in CD38+CD16- NK cells remains unknown.

CD16 expression could explain why CD38+ NK cells play opposite roles in the immunoregulation of CRC and RA. CD16 is also termed low-affinity immunoglobulin gamma Fc region receptor III-A. It is expressed on the NK cell surface and acts as a receptor and a transmembrane peptide-anchored integral membrane glycoprotein. By inhibiting apoptosis of NK cell progenitors, CD16 specifically controls NK cell viability and growth (111). Interestingly, CD38 can downregulate CD16 expression in CD38+ NK cells from CRC patients because CD16 expression is increased after anti-CD38 antibody treatment of CD38+ NK cells (20). The surface proximity of CD38 and CD16 was demonstrated in cocapping experiments as well as in fluorescence resonance energy transfer experiments. Functional CD16 is a prerequisite for the ability of CD38 to regulate IFN-γ secretion, the tyrosine phosphorylation of zeta chain of T cell receptor associated protein kinase 70 (ZAP70) and a mitogen-activated protein kinase, and calcium flux and cytotoxicity. Furthermore, CD16 can restore the receptor function of CD38 in NK cells (43). Cell lines generated from NK cells that lacked CD16 could not mobilize Ca^2+^ via CD38. CD38 signaling can induce the release of IFN-γ and GM-CSF by NK cells expressing CD16 (112). The results of the above studies support the regulatory role of CD16 in CD38+ NK cells. To date, no data have shown how CD16 expression in CD38+ NK cells is correlated with HSPA1B, Sirt6 and NF-κB expression.

Although many studies have shown that the essential role of CD38 in NK cells is dependent on CD16, some studies suggest that both CD38+CD16+ NK cells and CD38+CD16- NK cells are involved in immunoregulation. One study reported that CD56_bright_CD16- NK cells produced ADO but suppressed autologous CD4+ T-cell proliferation. This inhibition was reversed by pretreating these NK cells with a CD38 inhibitor (103). Sialic acid-binding immunoglobulin-like lectin-7 (Siglec-7) is an inhibitory receptor and is preferentially expressed by mature NK cells from the peripheral blood of healthy adults. Siglec-7+ NK cells present reduced levels of the inhibitory receptors NKG2A and CD158b but increased levels of the activating receptors CD16, CD38, NKp30, DNAX accessory molecule-1 (DNAM1) and NKp46. Moreover, compared with Siglec-7+ NK cells, Siglec-7− NK cells produce less IFN-γ and CD107a (113). CD38+CD16+ NK cells and CD38+CD16- NK cells are likely related to Siglec-7− NK cells and Siglec-7+ NK cells, respectively.

**Figure 3:**
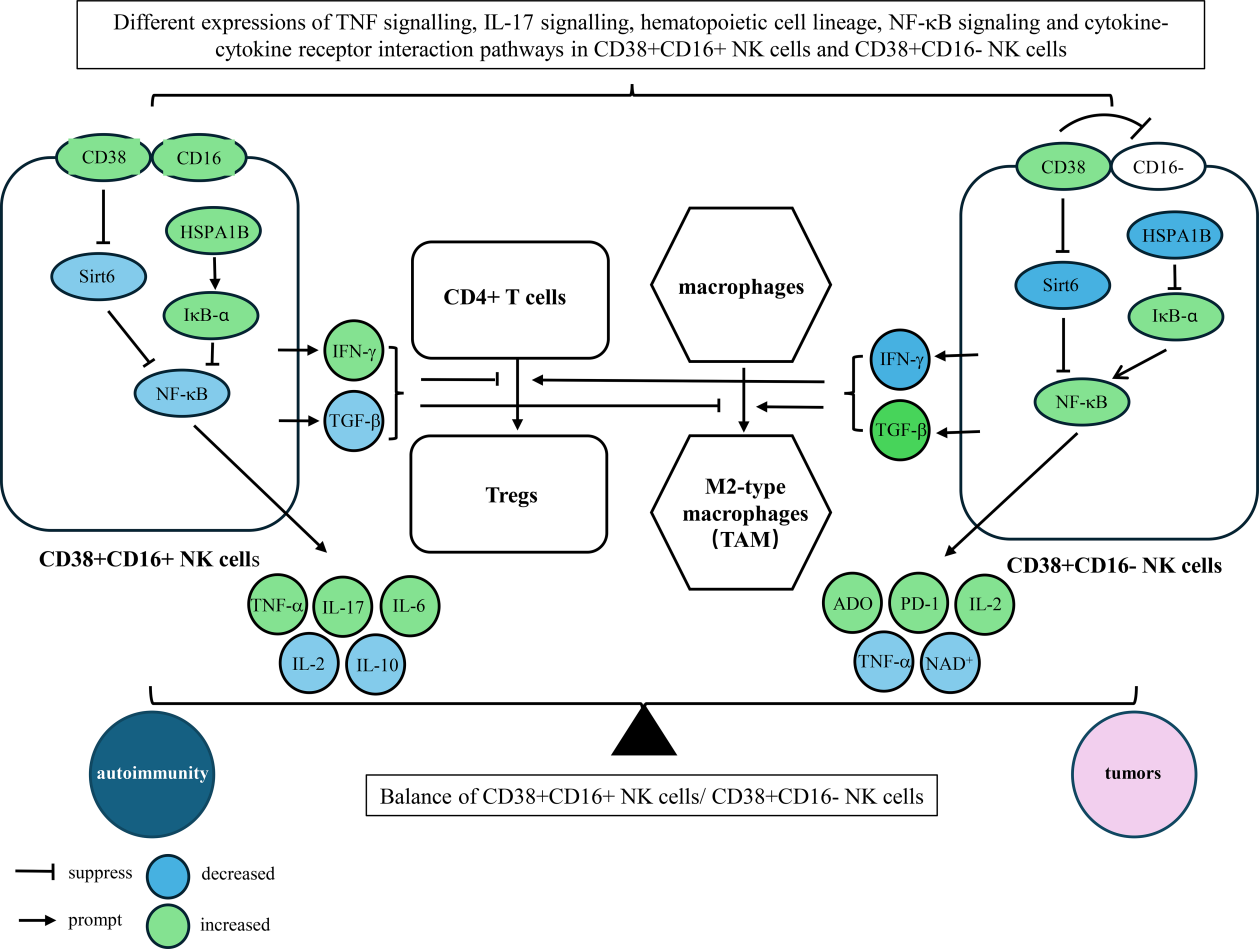
Potential mechanism through which CD38+ NK cells regulate immune balance. The CD38+CD16+ NK cell/CD38+CD16- NK cell ratio affects immune balance and mediates immune surveillance and immune tolerance by regulating proinflammatory cytokine, ADO and NAD+ production as well as macrophage polarization and CD4+ T-cell differentiation.

The differences in function and regulatory mechanisms between CD38+CD16+ NK cells and CD38+CD16- NK cells are proposed in **Figure 3** and **Table 2**.

**Table 2:** Comparison of the key molecules in CD38+NK cells.

Key molecule and mechanism	CD38+ NK cells from RA and healthy people	CD38+ NK cells from CRC
CD38	high expression	high expression
CD16	expressed	low expression
Sirt1/Sirt6	low expression	low expression
HSPA1B	high expression	low expression
NF-κB	low expression	high expression
NAD+		low expression
ADO		high expression
PD-1		high expression
IFN-γ	high expression	low expression
TGF-β	low expression	high expression
TNF-α	high expression	low expression
IL-6	high expression	
IL-2	low expression	
IL-10	low expression	
CD4+T cell differentiation to Tregs	inhibited	stimulated
macrophage polarization to M2 subtype		stimulated
killing activity		low

To date, the evidence for immunoregulation of CD38+ NK cells is still preliminary. The inflammatory and killing potential of the CD38+CD16+ NK cell subtype could be inhibited in the tumor microenvironment, regardless of the proportion or number of CD38+CD16- NK cells present. The function of CD38+CD16+ NK cells may vary depending upon their location and tissue environment. In addition, Tregs adversely affect the activation of NK cells in lymph nodes, thus promoting lymph node metastasis (114). Tregs hinder the homeostatic proliferation of NK cells, the cytotoxic activity of NKG2D-dependent NK cells, and IFN-γ secretion by IL-12–induced NK cells (84). M2 macrophages from the peritoneum and bone marrow also inhibit the activation of NK cells and their cytotoxicity against tumor cells (115). Moreover, some studies have reported that CD38+ MDSCs are also involved in immunoregulation in patients with CRC and in a murine model of esophageal cancer (116, 117). In the tumor microenvironment, various factors are intertwined and complicated, and it is not certain that CD38+ NK cells play an absolute role in immunoregulation.

## 6 Diagnostic and therapeutic implications of CD38+ NK cells

As described above, compared with healthy controls (9.71 ± 3.40%), patients with RA had a higher percentage of CD38+ NK cells (CD38+CD3−CD16+CD56+ cells) among total lymphocytes (CD45+ lymphocytes) (13.08 ± 5.04%) (*P* = 0.0037). Moreover, the CD38+ NK cell/CD38+ NK-like T cell (CD38+CD3+CD16+CD56+ cells) ratio ranged from 0.62 to 23 (mean: 8.34) in RA patients and from 0.31 to 11.26 (mean: 1.153) in healthy controls. Nonetheless, the NK cell/NK-like T-cell ratio did not differ between patients and healthy individuals (*P* = 0.9227). Furthermore, a positive correlation was detected between the CD38+ NK cell/CD38+ NK-like T-cell ratio and disease activity score in 28 joints (DAS28 score) in the patients (r = 0.716, *P* < 0.0001) (42). Therefore, the CD38+ NK cell/CD38+ NK-like T-cell ratio could be considered for the clinical diagnosis and prognosis evaluation in patients with RA. On the other hand, the percentages of CD38+CD16- NK cells among total lymphocytes in blood samples from patients with GC, CRC, OC, and LC were significantly greater than those of controls. Additionally, the CD38+CD16+ NK cell/CD38+CD16- NK cell ratio was significantly lower in blood samples from patients with LC (mean: 22-fold), CRC (mean: 40-fold), and OC (mean: 22-fold) than in healthy control samples (mean: 60-fold) (20). Therefore, the CD38+CD16+ NK cell/CD38+CD16- NK cell ratio in the peripheral blood of tumor patients could be evaluated for clinical diagnosis and prognosis and even for immune state assessment.

C3G has been reported to possess antioxidant, anti-inflammatory, and antitumor properties (118–120). It also protects against neurodegenerative diseases (121, 122). Additionally, C3G exerts antiaging effects via CD38-Sirt6 signaling (123). C3G can mitigate the CD38+ NK cell-mediated suppression of CD4+ T cell differentiation into Tregs. Thus, C3G may have therapeutic benefits for treating RA and CIA (33). Some studies have reported that 78c reduces the neuroinflammation of astrocytes and microglia caused by increased NAD^+^ levels (124). 78c also protects the heart against postischemic injury (35). Additionally, 78c improved the health span and longevity in a mouse model of chronological aging by reversing the reduction in tissue NAD^+^ levels and subsequently alleviating age-related metabolic dysfunction (36, 125). 78c treatment can increase NAD^+^ levels in the liver and muscles (126). Our study showed that 78c had a therapeutic effect on CIA mice. Furthermore, the inhibitory effect of CD38+ NK cells on the differentiation of CD4+ T cells into Tregs was considerably reduced by 78c. Hence, similar to C3G and anti-CD38 antibodies, 78c may be used to treat RA (33, 37).

## 7 Limitations and open questions about CD38+ NK cells

Owing to the limitations of cell separation technology, the CD38+ NK cells used in the functional experiments were obtained from the peripheral blood of cancer patients rather than from their solid tumor tissues. Thus, it can only be inferred that this NK cell subtype from peripheral blood plays a role similar to that of the NK cell type from tumor tissues in the immune microenvironment. Moreover, the numbers of CD38+CD16- NK cells were insufficient to compare their effects with those of CD38+CD16+ NK cells in the *in vitro* experiments. It is believed that with advancing cell separation technology, related studies will be performed in this area.

Currently, no studies have directly compared the differences in immune regulatory mechanisms between CD38+CD16+ and CD38+CD16- NK cells in tumors or autoimmune diseases. Such studies in tumor-bearing mice, CIA mice or *in vitro* cultured NK cells are also lacking. This is an important area of future research. Comparative experiments are needed to obtain direct evidence and identify the differences in cellular function and molecular mechanisms between CD38+CD16+ and CD38+CD16- NK cells, especially in terms of the differential expression levels of NF-κB, HSPA1B, IFN-γ, TNF-α, IL-2, TGF-β, ADO and NAD^+^. The mechanisms by which CD38+CD16- and CD38+CD16+ NK cells affect CD4+ T-cell differentiation and macrophage polarization must be thoroughly investigated. Single-cell sequencing can be applied to examine cultured CD38+ NK cells and tumor tissues. The effects of 78c, anti-CD38 antibodies and C3G, as well as Sirt6 and NF-κB inhibitors and activators, should also be explored by analyzing the two NK cell subtypes.

To date, few studies have investigated CD38- NK cells (CD38-CD3-CD56+ cells). Compared with CD38+ NK cells, CD38_low_/- NK cells exhibit a noticeably greater capacity for proliferation, whereas CD38+ NK cells display greater cytotoxicity against MM cells (19). When daratumumab, an antibody that targets surface CD38 for MM treatment, was used, the elimination of MM cells was more successful with CD38_low_/- NK cells because of their cytotoxicity than with CD38+ NK cells (19, 127).

NK cell therapy has been proven to be effective in treating cancer. At present, there are no precision or personalized treatment strategies for patients according to the subtypes of NK cells used during cell therapy. On the basis of our ongoing work, NK cells derived from umbilical cord blood and peripheral blood have different subtypes and expression profiles; thus, their therapeutic effects can differ across tumor patients. Even NK cells from the peripheral blood of different donors have different therapeutic effects. Many people who have a history of autoimmune disease have more CD38+CD16+ NK cells than healthy people do; moreover, tumor patients may have more CD38+CD16- NK cells. However, NK cells extracted and expanded from peripheral blood of these patients are often clinically used for self-treatment or treatment of their relatives.

In summary, studies over the past 20 years have highlighted CD38+ NK cells as a novel NK cell subtype. CD38+CD16+ and CD38+CD16- NK cells may cooperate to regulate immune tolerance and immune surveillance. The above data may be helpful for understanding the mechanisms underlying the disruption of immune balance.
